# Decoding the perception of pain from fMRI using multivariate pattern analysis

**DOI:** 10.1016/j.neuroimage.2012.08.035

**Published:** 2012-11-15

**Authors:** Kay H. Brodersen, Katja Wiech, Ekaterina I. Lomakina, Chia-shu Lin, Joachim M. Buhmann, Ulrike Bingel, Markus Ploner, Klaas Enno Stephan, Irene Tracey

**Affiliations:** a Centre for Functional Magnetic Resonance Imaging of the Brain (FMRIB), Nuffield Department of Clinical Neurosciences, Nuffield Division Anaesthetics, University of Oxford, John Radcliffe Hospital, Oxford OX3 9DU, UK; b Translational Neuromodeling Unit (TNU), Institute for Biomedical Engineering, University of Zurich & ETH Zurich, Wilfriedstrasse 6, CH 8032 Zurich, Switzerland; c Department of Computer Science, ETH Zurich, Universitaetstrasse 6, CH 8092 Zurich, Switzerland; d NeuroImage Nord, Department of Neurology, University Medical Center Hamburg-Eppendorf, Martinistr. 52, 20246 Hamburg, Germany; e Department of Neurology, Technische Universität München, 81675 Munich, Germany; f Wellcome Trust Centre for Neuroimaging, University College London, UK

**Keywords:** Pain, Decoding, Support vector machine, Permutation test, Classification accuracy

## Abstract

Pain is known to comprise sensory, cognitive, and affective aspects. Despite numerous previous fMRI studies, however, it remains open which spatial distribution of activity is sufficient to encode whether a stimulus is perceived as painful or not. In this study, we analyzed fMRI data from a perceptual decision-making task in which participants were exposed to near-threshold laser pulses. Using multivariate analyses on different spatial scales, we investigated the predictive capacity of fMRI data for decoding whether a stimulus had been perceived as painful. Our analysis yielded a rank order of brain regions: during pain anticipation, activity in the periaqueductal gray (PAG) and orbitofrontal cortex (OFC) afforded the most accurate trial-by-trial discrimination between painful and non-painful experiences; whereas during the actual stimulation, primary and secondary somatosensory cortex, anterior insula, dorsolateral and ventrolateral prefrontal cortex, and OFC were most discriminative. The most accurate prediction of pain perception from the stimulation period, however, was enabled by the combined activity in pain regions commonly referred to as the ‘pain matrix’. Our results demonstrate that the neural representation of (near-threshold) pain is spatially distributed and can be best described at an intermediate spatial scale. In addition to its utility in establishing structure-function mappings, our approach affords trial-by-trial predictions and thus represents a step towards the goal of establishing an objective neuronal marker of pain perception.

## Graphical abstract

**Figure:**
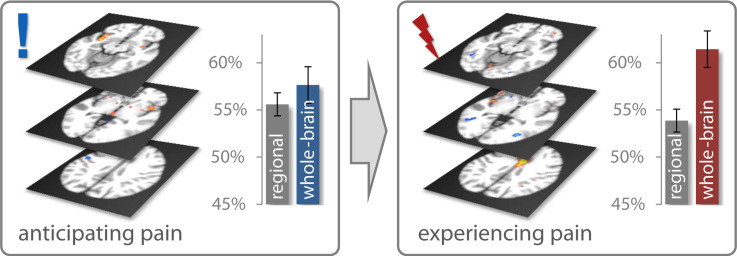

**Highlights:**

► Subjects received a series of near-threshold pain stimuli while undergoing fMRI. ► We studied multivariate patterns of brain activity underlying pain perception. ► PAG, VLPFC, DLPFC, anterior insula, and OFC were highly predictive of pain. ► Yet the highest accuracies were afforded by combinations of these regions. ► This suggests that pain is encoded in a distributed network.

## Introduction

The perception of pain is a multi-factorial experience that comprises sensory, cognitive, and affective aspects. Accordingly, pain is thought to result from a complex interplay between many regions in the human brain, including the thalamus, insula, primary and secondary somatosensory, anterior cingulate cortex, and prefrontal cortex (Apkarian et al., 2005). The specific characteristics of regions underlying the perception of pain have been described in some detail using conventional univariate analysis methods for functional magnetic resonance imaging (fMRI). By contrast, there have been almost no attempts at examining the distributed representation of pain and how it is encoded jointly by activity within and across the set of regions commonly associated with pain.

Statistical methods for examining distributed coding schemes have undergone rapid progress over the past years. One particularly versatile approach, termed multivariate pattern analysis (MVPA), is based on the use of a classification algorithm to infer a perceptual or cognitive state from brain activity. The underlying multivariate decoding models differ in important ways from univariate encoding models, such as the general linear model (GLM). Univariate analyses have proven powerful for inference on structure-function mappings in the brain when activations are expressed in terms of local peaks or clusters of activity (Friston et al., 1995). However, they are less suitable for assessing the amount of information encoded in spatially distributed (multivoxel) patterns of activity underlying specific perceptual or cognitive states. This information can be estimated using multivariate decoding models (Friston et al., 2008; Haynes and Rees, 2006; Norman et al., 2006; O'Toole et al., 2007; Pereira et al., 2009). These models consider several voxels at the same time and may therefore be more sensitive than univariate models (for an analysis of the conditions under which this is the case, see Guyon and Elisseeff, 2003).

Decoding approaches are typically implemented in the form of classification algorithms. The results of such algorithms are often reported in terms of classification accuracies. It is important to remember, however, that in cognitive neuroscience the absolute accuracy is not of primary interest if, as we do here, one wishes to demonstrate the existence of a structure-function relationship in the brain, e.g., the relationship between measures of brain activity and a perceptual state (Friston et al., 2008). Such a relationship is evidenced by the *significance* with which the accuracy is above chance, not by its *magnitude*, since the significance takes into account both mean and variability in the group. This is different in engineering applications such as the design of brain-machine interfaces, where *substantive* significance, i.e., the magnitude of classification accuracy, is of interest. Thus, inferences in this paper are not based on accuracies but on the question of whether the reported accuracies are significantly above chance; similarly, comparisons are not based on absolute differences in accuracies but on the question of whether two accuracies differ significantly. We will revisit this distinction in the Discussion.

The utility of classification approaches has been demonstrated in many domains of systems neuroscience, but corresponding insights into the perception of pain have remained scarce. In one methodological study, the utility of Gaussian processes was illustrated using different levels of pain as well as graded responses to similar levels of pain (Marquand et al., 2010). Another technical study considered the temporal evolution of perception in response to prolonged noxious stimulation (Prato et al., 2011).

These studies have suggested that predicting pain from brain recordings may be feasible. However, it has remained unclear to what extent the extraction of pain-related information benefits from the simultaneous consideration of multiple brain regions. More specifically, it is not well understood which spatial scale^2^ is optimal for decoding pain: individual voxels, single anatomical regions, combinations of regions, or whole-brain activity? Moreover, it is currently unknown what predictive capacity is enabled by those anatomical regions (and their combinations) that are typically associated with pain. Finally, there has been no investigation of pain encoding that assesses voxel-wise significances (e.g., *t*-scores) in a multivariate fashion.

In the present study, we addressed the above questions by analyzing the predictive capacity of individual and multiple brain regions in decoding the subjective experience of pain. Notably, we carry out this analysis in the setting of rather subtle (near-threshold) pain stimuli. This is challenging but important since decoding results may otherwise be dominated by physical differences in sensory stimulation rather than differences in subjective pain experience. First, we aimed to predict pain perception from whole-brain fMRI data on a trial-by-trial basis. Second, we examined which spatial level of description enabled the most accurate predictions of pain: single voxels, individual anatomical regions, combinations of regions, or whole-brain activity. For both questions, we trained and tested a linear support vector machine (SVM) on trial-specific correlates of whole-brain activity using a leave-one-session-out cross-validation scheme. Third, we evaluated SVM-based voxel weights with a permutation test to illustrate the spatial deployment of jointly informative voxels throughout the brain.

## Methods

### Participants

To study the multivariate nature of pain-related activity in the brain, we revisited a dataset that was originally analyzed using conventional univariate methods (Wiech et al., 2010). Here, we provide a summary of the underlying experimental design, focusing on those aspects that are relevant for the question addressed in the present paper. A group of 16 volunteers (age range 19–30 years, 11 females, all right-handed), with no history of neurological or psychiatric illnesses or chronic pain, participated in the study. All participants gave informed consent, and the study was approved by the local Research Ethics Committee.

### Experimental design

**Fig. 1:**
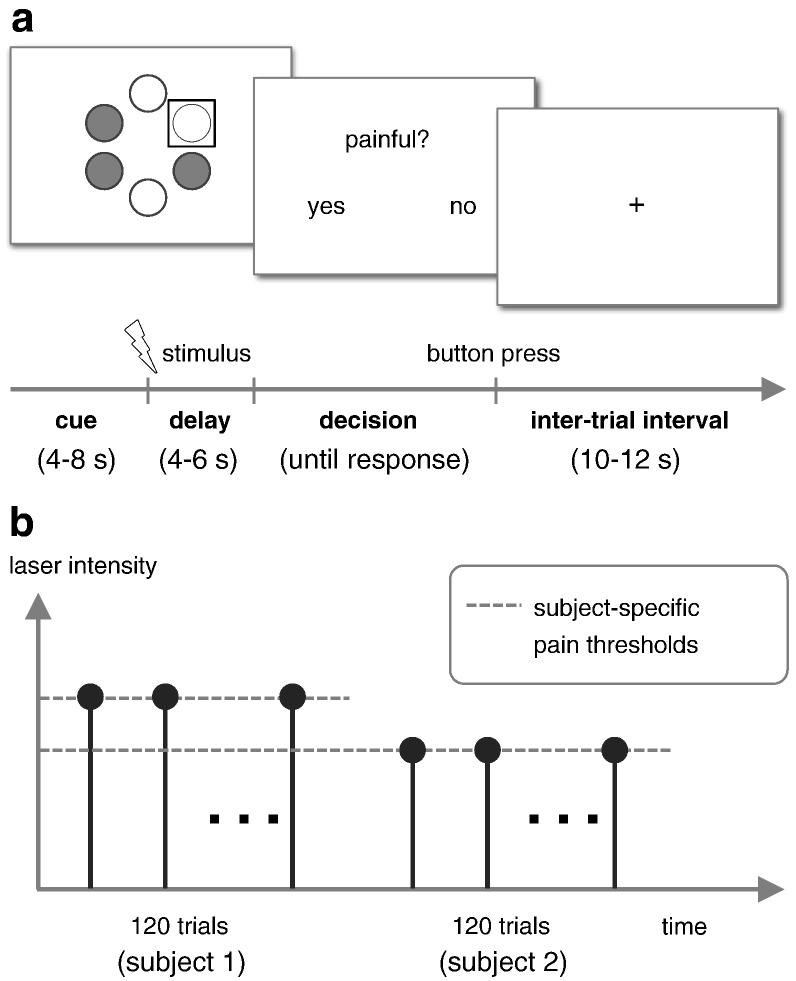
Experimental design. Subjects were engaged in a simple perceptual decision-making task (Wiech et al., 2010). (a) At the beginning of each trial, a graphical representation of the 6 potential stimulation sites was shown before stimulus application. ‘Fully approved’ sites were shown in a different color than sites that were ‘approved with reservations.’ The site stimulated on the current trial was highlighted by a square. Following a brief laser stimulus, participants were prompted to indicate by a button press whether the stimulus had been perceived as painful (here: left button for ‘pain’, right button for ‘no pain’). Assignment of buttons was randomized across all 120 trials. (b) Within each subject, the laser intensity was calibrated to match the individual pain threshold.

Subjects were engaged in a sensory decision-making task consisting of carefully calibrated laser stimulation and an additional threat manipulation (Fig. 1). The experiment consisted of four sessions, each comprising 30 trials, totaling 120 trials per subject. On each trial, a near-threshold laser stimulus was applied to one out of six possible stimulation sites on the right foot. Following the laser pulse, participants were prompted to indicate by button press whether the stimulus had been perceived as painful or non-painful.

The design contained an additional factor which was of no interest in the present analysis, but whose details we briefly outline for completeness (see Wiech et al., 2010, for a full description). At three stimulation sites, participants were made to believe that the stimulation was safe and approved without reservations (‘low threat’ condition). At the remaining three sites, participants were told that the stimulation would still be performed but could only be approved with reservations, as a result of an assessment of skin properties prior to the experiment (‘high threat’ condition). Unknown to participants, the assignment of the six sites to the two conditions (low threat vs. high threat) was defined a priori and entirely unrelated to any actual skin properties. On each trial, a visual cue informed subjects whether the laser stimulus was about to target a ‘low threat’ or a ‘high threat’ site.

### Data acquisition and preprocessing

Using a 3T MRI scanner (Oxford Magnet Technology, Oxford, UK), whole-brain functional T2*-weighted echo-planar images (EPI) were acquired with BOLD contrast (TR 3 s; TE 30 ms; flip angle 90°; matrix 64 × 64; field of view 192 mm × 192 mm; 41 axial slices; slice thickness 3 mm). The first 4 volumes were discarded to compensate for T1 saturation effects. Using SPM8 (http://www.fil.ion.ucl.ac.uk/spm), images were realigned to the first volume and unwarped. Images of all sessions were spatially normalized to the standard EPI template included in SPM, using a fourth-degree B-spline interpolation.

### Univariate analysis

Prior to the classification-based analyses described below, we performed several conventional univariate analyses for comparison. For these analyses, images were spatially smoothed with an isotropic Gaussian kernel (FWHM 8 mm). First, we investigated the main effect of pain during anticipation and stimulation. To this end, we constructed a (first-level) GLM for each subject with a design matrix that included separate ‘pain’ and ‘no pain’ regressors for the anticipation and the stimulation period (4 regressors), collapsing across ‘low threat’ and ‘high threat’ trials, whose distinction was of no interest in the present study. Anticipation periods were modeled according to their trial-specific durations (i.e., 4–8 s), while the stimulus duration was modeled as 1 s. Serial autocorrelation and low-frequency drifts were accounted for using a first-order autoregressive model and a high-pass filter (cut-off 128 s), respectively. Group-level inferences for the anticipation and stimulation period were made by entering the appropriate contrast into an ANOVA, using the following two contrasts: (1) pain vs. no pain during anticipation; and (2) pain vs. no pain during stimulation.

### Multivariate analysis

In contrast to univariate analyses, multivariate approaches explicitly account for dependencies between voxels, which allows for inference on distributed responses. In this study, we trained and tested a linear support vector machine (SVM) on trial-wise fMRI data. In order to avoid a potential bias resulting from serial autocorrelations, we used leave-one-session-out cross-validation. Specifically, we trained an SVM on trials from three sessions and tested it on trials from the fourth (left-out) session, repeating this process four times. To obtain trial-wise data for classification, we constructed a GLM with a design matrix that included separate boxcar regressors for the anticipation phase and the stimulation phase of each individual trial (240 regressors). We used this GLM as a filter to obtain separate parameter-estimate images (beta images) for the anticipation phase and the stimulation phase of each trial. These images were processed further in two ways. First, we standardized the parameter estimates within each voxel (implying mean = 0 and standard deviation = 1). Second, we scaled all images such that within each trial the $l_{2}$-norm of parameter estimates became 1. The resulting images were used in two sets of classification analyses, as described next (for a structured list of individual analysis steps, see Section C in the Supplemental Material).

In the first analysis, we investigated whether fMRI data contained sufficient information to predict, on a trial-by-trial basis, the perception of pain. For this purpose, a linear SVM was trained and tested on different anatomical scales. These independent analyses were based on (i) the single most discriminative voxel (which was determined using a *t*-contrast as described in the second analysis below, and whose identity was allowed to vary both between cross-validation folds and between subjects), (ii) combinations of discriminative voxels (i.e., differently sized groups of voxels that were individually discriminative, as determined using a *t*-contrast as described below), (iii) single anatomical regions typically associated with pain processing (see below), (iv) combinations of the most predictive anatomical regions, and (v) whole-brain data.

Within each cross-validation fold, we used another (nested) level of (leave-one-trial-out) cross-validation on the training data to optimize the regularization hyperparameter *C*. In this way, test data were neither used for training nor for the optimization of hyperparameters, guaranteeing a non-circular analysis. Furthermore, to ensure that the analysis was not confounded by differences between ‘high threat’ and ‘low threat’ trials, we ran two separate decoding analyses on the two trial types and considered the mean accuracy. This procedure was repeated for every subject to obtain an estimate of mean classification accuracy in the group (cf. Section C in the Supplement).

We used a nonparametric permutation test to evaluate the null hypothesis that there was no statistical link between fMRI data and the perception of pain. This null hypothesis corresponds to a mean population accuracy at the level of chance (i.e., 0.5). Thus, we repeated each classification analysis *N* times using labels that were randomly permuted within sessions, preserving the assumption of exchangeability underlying the permutation test for our leave-one-session-out cross-validation scheme. For each analysis, we computed a *p*-value as: the rank of the original sample accuracy in the distribution of permutation-based sample accuracies, divided by the number of permutations. We generally used *N* = 1000. In the case of ROI-specific analyses with their 26-fold multiple-comparison correction, we used *N* = 2600 to allow for the detection of significance at the 0.05 level (see below).

In the second analysis, we characterized the spatial deployment of jointly informative voxels across the brain by combining an SVM with a permutation test on voxel weights (LaConte et al., 2005; Mourao-Miranda et al., 2005). Specifically, we trained a linear SVM on whole-brain data (using all 120 trials in each subject) and reconstructed the spatial deployment of voxel-wise weight coefficients. These coefficients may heavily depend on task-unrelated sources of variance in the data and are generally not interpretable as such. One way of addressing this issue is to relate voxel weights to their empirical null distributions, i.e., those distributions that one would obtain if no statistical relationship between BOLD activity and pain perception existed (Mourao-Miranda et al., 2005; Wang et al., 2007).

To obtain these distributions, we randomly permuted trial-specific labels and re-estimated the model based on the new labels. Unlike in the case of all other multivariate analyses presented in this paper, a *nonparametric* permutation is computationally intractable if one wishes to obtain a whole-brain FWE-corrected map with fine-grained discriminability eve among top-scoring voxels. For this particular analysis, we therefore resorted to a *parametric* approach. Using a Kolmogorov–Smirnov test (test size α = 0.05), we found that less than 0.01% of all voxel-specific null distributions were not Gaussian. Thus, we summarized each null distribution in terms of the mean and variance of a Gaussian. Using these null distributions, we evaluated the probability with which the weight *w*_*v*_ in voxel *v* would have been observed under the null. Formally, this test is based on a *t*-score, defined as

$$t_{v}=\frac{w_{v}-{\hat{\mu }}_{v}}{{\hat{\sigma }}_{v}}\sim t_{N-1}\text{,}$$

where ${\hat{\mu }}_{v}$ and ${\hat{\sigma }}_{v}$ denote the sample mean and standard deviation of voxel weights in voxel *v* across all random permutations, and *t*_*N* − 1_ is Student's *t*-distribution on *N* − 1 degrees of freedom. We used *N* = 2000 permutations and corrected the resulting map for multiple comparisons using a conservative whole-brain family-wise error (FWE) correction (α = 0.05). This correction was based on the same application of random-field theory to estimate the smoothness of the data as was used for thresholding the (mass-univariate) SPMs (see above).

In summary, we obtained whole-brain FWE-corrected maps of *t*-scores by relating voxel-wise SVM weights to their null distributions, as obtained by repeatedly re-estimating the model based on permuted labels. In comparison to searchlight methods, this approach is of similar computational complexity but extends the search space from locally multivariate patterns to jointly informative patterns across the entire brain.

It is worth emphasizing that maps of voxel weights come with methodological limitations which may diminish their utility for drawing conclusions about the encoding of pain. In particular, the regions that are assigned the highest SVM weights may not necessarily be the regions that are most strongly related to pain responses. In addition, the above *t*-scores are based on the assumption that voxel weights are normally distributed under the null. Deviations from this assumption may be particularly severe in the far tails of the distributions, leading to inaccuracies among high *t*-scores. Thus, the spatial deployment of feature weights will serve illustrative purposes only, while conclusions about the representation of pain will first and foremost be based on regional analyses of classification accuracy, as described next.

### Region-of-interest analysis

Using published reference atlases (Harvard–Oxford cortical and subcortical structural atlases, Harvard Center for Morphometric Analysis; Jülich Histological Atlas, Research Centre Jülich) and individual T1 images, we defined 26 masks for pain-related brain regions (see Fig. S1 and Section B in the Supplemental material). We trained and tested a linear SVM independently on these ROIs and evaluated the resulting performance in the same way as described before (using nonparametric permutation tests; see ‘Multivariate analysis’). Since this analysis was carried out independently for each ROI, we controlled the family-wise error by correcting significance thresholds for multiple testing using a conservative Bonferroni correction based on the number of regions. In order to reduce the potentially confounding effect of smoothing on the comparison between voxel-based and ROI-based feature selection, we used a smaller smoothing kernel (FWHM 5 mm) in these analyses.

## Results

### Decoding pain from whole-brain data

Asking whether subtle trial-by-trial variations in distributed brain activity could be used to predict perceptual decisions about pain, we trained and tested a linear SVM on trial-wise correlates of fMRI data. We found that whole-brain activity both *before* (57.6%) and *during* (61.4%) the application of a near-threshold laser stimulus enabled predictions significantly above chance level (*p* < 0.001; nonparametric permutation test using *N* = 1000 permutations).

For completeness, we tested which subjects showed effects that were significant even when considered in isolation (see Supplemental Fig. S1). We found that 1 out of 16 subjects by itself showed an accuracy that was significantly above chance in the anticipation phase, and 3 out of 16 in the stimulation phase (*p* < 0.05; nonparametric permutation test using *N* = 1000; Bonferroni-corrected).

Though not of interest in the present study, we also examined whether the condition (‘threat’ vs. ‘no threat’) could be predicted from trial-wise fMRI data, in particular in the anticipation phase in which the condition was disclosed on the screen; we obtained mean classification accuracies of 55.7% (*p* < 0.05) before and 52.2% (n.s.) during the stimulation.

**Fig. 2:**
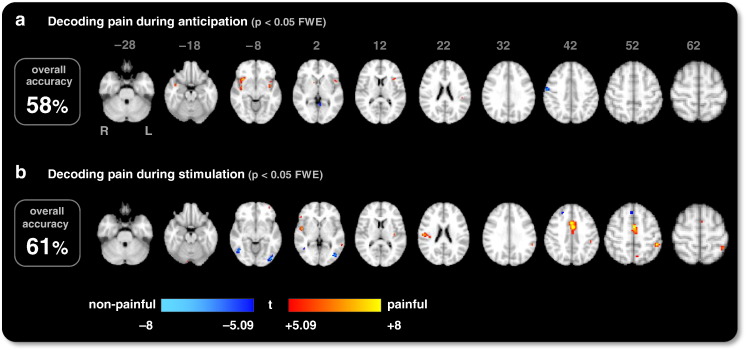
Pain perception in individual voxels. This discriminative map shows the statistical significance of voxel weights obtained by training a linear support vector machine (16 subjects). Separate analyses were conducted based on brain activity (a) *before* and (b) *during* stimulus application. Regions highlighted in red represent voxels whose activity is higher on subjectively painful trials than on non-painful trials, whereas blue regions represent voxels whose activity is higher on non-painful trials than on painful trials (*p* < 0.05, FWE-corrected, see Methods section). Results are overlaid onto a standard structural scan in MNI152 space. The percentages on the left indicate the resulting classification accuracies when using a whole-brain feature space. Both are significantly above chance (*p* < 0.001; see main text).

In order to provide an intuition about which data features were responsible for above-chance accuracies in predicting pain, we examined the spatial deployment of voxels that played an important role in classification. To this end, we adopted an exploratory perspective and used an SVM with a permutation test on feature weights to map discriminative information continuously throughout the brain. This approach highlights those voxels that are most influential in shaping the separating hyperplane used by the SVM to distinguish between painful and non-painful trials (Fig. 2). In contrast to other commonly used multivariate techniques, such as searchlight classification methods that are locally multivariate, our approach simultaneously considers all voxels in the brain.

We found that in the anticipation period, the bilateral insula proved most predictive for the perception of pain. In the stimulation period, brain activity in the mid cingulate cortex (MCC), SI/SII, bilateral insula and orbitofrontal cortex (OFC) allowed for significant predictions of the perception of pain (for a comparison with a conventional mass-univariate map, see Fig. S2 in the Supplemental material).

### Decoding pain from individual regions of interest

**Fig. 3:**
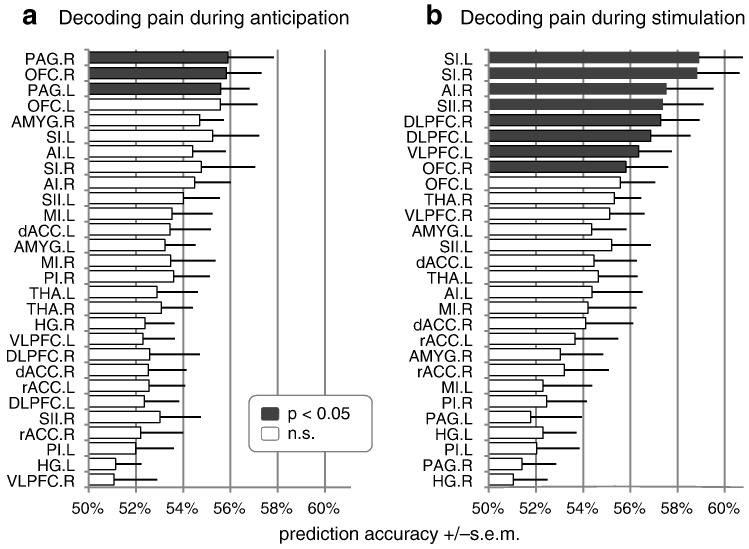
Pain perception in individual regions of interest. The figure shows prediction accuracies obtained in 26 regions of interest for the differentiation between trials experienced as painful or non-painful (a) *before* and (b) *during* stimulation (plus two control regions, HG.L and HG.R). Results are given in terms of mean accuracy +/− standard error of the mean, based on 16 subjects. Statistical inference is based on a nonparametric permutation test with *N* = 2600 permutations and Bonferroni correction for multiple testing. Note that regions are sorted by the significance of prediction accuracies (*p*-values), not by their magnitude. AI/MI/PI = anterior/mid/posterior insula; AMYG = amygdala; dACC/rACC = dorsal/rostral anterior cingulate cortex; DLPFC/VLPFC = dorsolateral/ventrolateral prefrontal cortex; HG = Heschl's gyrus; OFC = orbitofrontal cortex; PAG = periaqueductal gray; SI/SII = primary/secondary somatosensory cortex; THA = thalamus; *.R = right; *.L = left.

Having predicted pain perception based on whole-brain activity, we next asked whether it is possible to predict pain perception based on fMRI activity within individual brain regions. To investigate this, we attempted to decode the anticipation and perception of pain from 26 predefined brain areas typically reported in the context of pain (plus 2 control areas). It should be noted that these regions were defined a priori on anatomical grounds, independently from the results of the above whole-brain analysis. We assessed the importance of each region in terms of the significance with which it enabled above-chance predictions. This approach allowed us to propose a rank order of pain-related regions. Critically, this rank order is based on significance rather than accuracy, and thus takes into account not only the mean accuracy but also its between-subjects variability (Fig. 3).

It should be noted that the whole-brain analysis in the previous section and the region-of-interest analysis in this section are based on different notions of involvement. Thus, one would expect their results to share the most important, but not necessarily all, characteristics. We will expand on this point in the Discussion.

Of all brain regions commonly associated with the perception of pain, our analysis revealed that only a subset was predictive of pain on a trial-by-trial basis. The most predictive regions during the *anticipation period* were the right and left periaqueductal gray (PAG) and right orbitofrontal cortex (OFC; all *p* < 0.05; permutation test using *N* = 2600; Bonferroni-corrected for multiple testing across ROIs; Fig. 3a). During the *stimulation period*, the most predictive regions were the right and left primary somatosensory cortex (SI), right anterior insula, right secondary somatosensory cortex, right and left dorsolateral prefrontal cortex (DLPFC), left ventrolateral cortex (VLPFC), and right OFC (all *p* < 0.05; *N* = 2600; corrected; Fig. 3b). In terms of magnitudes, in the anticipation period, the highest classification accuracy was afforded by the right PAG, while the most accurate predictions for the stimulation period were enabled by activity in the left SI. By contrast, no above-chance performance was obtained when using gray-matter control masks of regions not involved in pain processing (left and right Heschl's gyrus, HG.L and HG.R).

It should be kept in mind that all of the above numbers are sample accuracies which serve as estimates of their corresponding unknown population mean accuracies. The uncertainty associated with these estimates (i.e., their standard error) is non-negligible since it reflects the between-subjects variability in the population (i.e., random effects). The rank order of regions is particularly sensitive to this variability and should therefore only be interpreted as an approximate guide to true differences in regional informativeness.

One finding, perhaps surprising at first, was the lack of above-chance prediction accuracy obtained in the signal of the mid and posterior insula during stimulation. This would be in contrast to previous findings in which both insula subdivisions have been implicated in stimulus-dependent processing (Albanese et al., 2007; Raij et al., 2005; Singer et al., 2004). The posterior insula in particular is considered a key region for nociceptive processing (Oertel et al., 2011). It is conceivable that this seeming discrepancy is a result of the way in which our anatomical masks were defined. As highlighted before, all regions of interest were defined on the basis of anatomical landmarks, not functional contrasts, and all voxels within a given area entered the respective multivariate analysis. Some regions, such as the posterior insula, are known to be somatotopically organized (Brooks et al., 2005), suggesting that predictions from the entire region might not faithfully reflect the impact of a particular somatotopically relevant subregion. Under this view, the surprisingly low accuracies in the posterior insula might be a consequence of the functional heterogeneity across anatomical subdivisions included in our anatomical mask.

We tested this hypothesis in an additional *post-hoc* analysis in which we utilized results from a previous study investigating the somatotopic organization of the insula (Brooks et al., 2005) to consider those portions of the posterior insula that had been implicated in the processing of somatosensory stimuli applied to the same site as in the present experiment (main effect of pain, N = 14, thresholded at *p* = 0.001). Contrary to our initial hypothesis, the prediction accuracy obtained in foot-specific portions of the (left) posterior insula was not significantly above chance during the stimulation period.

### Comparison of different spatial scales

As indicated above, activity patterns in several pain-related regions allow for the prediction of pain, both during anticipation and during stimulation. However, this does not necessarily imply that predictions become even more accurate when considering several brain regions simultaneously. To investigate whether this might be the case, we carried out two additional analyses in which we examined increasing spatial scales of encoding.

**Fig. 4:**
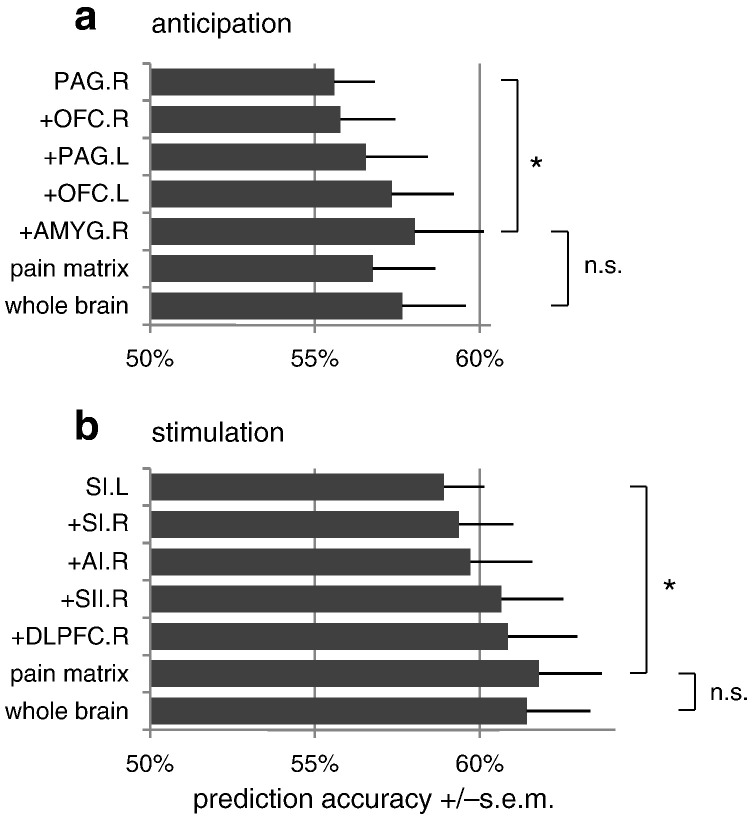
Pain perception in combinations of highly predictive regions. The figure shows prediction accuracies for the classification of painful versus non-painful trials, using different sizes of search space, (a) *before* and (b) *during* stimulation, based on 16 subjects. Results are given in terms of mean accuracy +/− standard error of the mean. All accuracies are significantly above chance (*p* < 0.01; nonparametric permutation test; *N* = 1000). Additional significances are indicated between accuracies on different sets of regions (*p < 0.05; permutation test).

In the first analysis, we focused on pain-related brain regions and tested whether prediction accuracies would benefit from increasing the search space (i.e., increasing the potential complexity of the model) from the most predictive single region to combinations of multiple regions (Fig. 4). In the anticipation period, the most predictive single ROI (i.e., the right PAG) yielded a prediction accuracy of 56% (*p* < 0.01; nonparametric permutation test using *N* = 1000; Fig. 4a). We found that this accuracy increased continuously when jointly considering additional regions and reached a significantly higher level (*p* < 0.05; *N* = 1000) when using the five most predictive regions (i.e., PAG, OFC, and right amygdala). Following this, the inclusion of additional regions did not yield further improvements in prediction accuracy, suggesting that the activation patterns in small combinations of regions, such as these five, encode sufficient complementary information to predict the perception of pain. Similarly, we observed no (significant) further improvement in prediction accuracy by moving to a whole-brain analysis.

These findings agreed nicely with the results that emerged from our analysis of the stimulation period. While being exposed to a stimulus, pain predictions from left SI, which was the most significant region, reached an accuracy of 59% (*p* < 0.01; nonparametric permutation test using *N* = 1000). Predictions from the combination of left SI and other brain regions that allowed for prediction of pain when considered in isolation (i.e., right SI, AI, SII, and DLPFC) did not lead to a significant increase in prediction accuracy. However, adding in the entire ‘pain matrix’ yielded a significant increase (*p* < 0.05; *N* = 1000) in prediction accuracy (to 62%) (Fig. 4b). This observation indicates that, while the overall accuracy is still well below 100%, the joint activation pattern of regions commonly summarized as the ‘pain matrix’ might enable the best predictions about pain perception that can be made on the basis of fMRI activity measures. Thus, both in the anticipation phase and in the stimulation phase, accuracies reached an optimum on the basis of a set of anatomical regions, with no further improvement enabled by considering whole-brain activity.

**Fig. 5:**
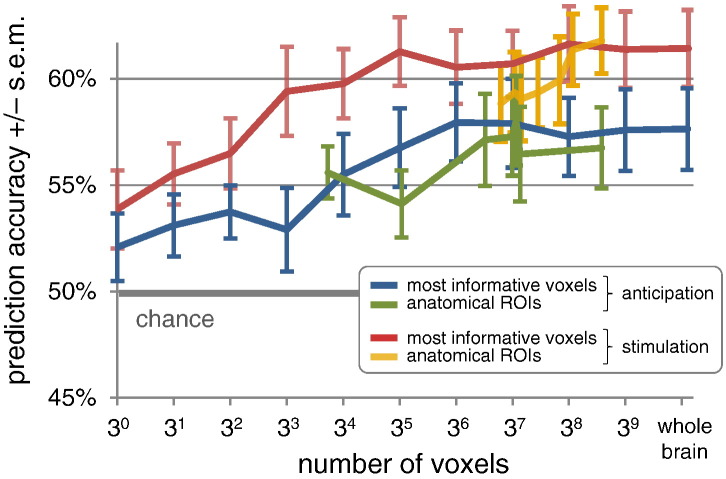
Pain perception across different scales. The two diagrams show the role of different spatial scales in relating brain activity to the perception of pain (a) *before* (blue) and (b) *during* (red) stimulus application, based on 16 subjects. Results are given in terms of mean accuracy +/− standard error of the mean. The gray horizontal bar indicates chance level (50%). All accuracies from 3^1^ (3) voxels onwards are significantly above chance (*p* < 0.05; nonparametric permutation test; *N* = 1000). For direct comparison of different strategies for feature selection, we imported the results from Fig. 4. Specifically, for each ROI shown in Fig. 4, we determined the number of voxels in the underlying anatomical mask (averaged across subjects). We then plotted the ROI-based accuracies at the corresponding locations on the x-axis (green and yellow lines).

In the second analysis, we examined a search space that was not based on anatomical regions of interest; instead, we considered individual voxels without anatomical constraints. We began with the single most predictive voxel, then tripled the number of voxels in each step, until the search space corresponded to a whole-brain analysis. Voxel-wise predictive strength was measured, independently within each cross-validation fold, in terms of *t*-scores, using a between-conditions two-tailed *t*-test. Using these discriminative scores, we found that the resulting prediction accuracies increased near-monotonically with the number of voxels considered, both in the anticipation and in the stimulation period, and leveled off towards the end (Fig. 5). Prediction of pain from the anticipation period increased from 52% (using a single voxel) to 58% (using all voxels; significantly above chance, *p* < 0.001; nonparametric permutation test; *N* = 1000). For the stimulation period, this accuracy increased from 54% (using a single voxel) to 61% (using all voxels, *p* < 0.001; *N* = 1000). Critically, the most rapid increase in accuracy was observed at small voxel numbers, while subsequent additions made very little contributions compared to the number of additional model parameters.

Overall, using a whole-brain search for the most informative voxels yielded higher prediction accuracies than the combinations of anatomical regions analyzed before. One might ask, of course, whether the two approaches could be compared on a finer scale. Here, we imported the results from Fig. 4 into Fig. 5. Specifically, for each ROI result shown in Fig. 4, we determined the corresponding position on the x-axis in Fig. 5 based on the region's number of voxels (averaged across subjects) and plotted its corresponding accuracy. We found that anatomical feature selection performed surprisingly well although it never significantly outperformed voxel selection based on the *t*-contrast.

## Discussion

The present study employed a multivariate decoding approach (i) to predict experiences of near-threshold pain from brain activity during the anticipation and receipt of pain, and (ii) to examine the distributed nature of pain perception. Our analysis led to three main findings.

First, we confirmed that it is possible to decode the perception of pain from trial-wise whole-brain fMRI data, even in the absence of physical stimulus alterations. In other words, we have demonstrated the existence of a subtle yet highly significant statistical link between measures of brain activity and pain perception. Second, while most brain regions commonly associated with pain allowed for above-chance decoding, these regions differed with regard to their predictive accuracy. Our analysis yielded a possible rank order of brain regions in which those regions turned out as most predictive that are generally considered critical for cognitive-affective pain processing or for sensory processing. Third, we found that fMRI activity on different spatial scales enabled different degrees of accuracy with which information about perceptions of pain can be decoded from fMRI data. Regarding the balance between accuracy (of the predictions) and complexity (of the model), a small set of anatomical regions of interest provided better explanations of subjective decisions about pain than individual voxels or individual regions.

### On the feasibility of predicting pain from brain activity

Multivariate analyses have been used to assess the predictability of a perceptual state from fMRI data in various contexts. The majority of existing studies have used classification algorithms to decode the identity of a physical stimulus (Haynes and Rees, 2006; Norman et al., 2006; O'Toole et al., 2007), where classifier predictions can be verified on the basis of the experimental design. First steps towards a different logic have been made by two recent methodological studies. One of them illustrated the utility of Gaussian processes by predicting both the identity of physically different stimuli and subjective responses to pain stimuli from the same class of intensity (Marquand et al., 2010). Another one explored the temporal evolution of the perceived intensity of prolonged pain stimulation (Prato et al., 2011).

In the present study, we focused specifically on the logic of constant-stimulus paradigms by using a design in which stimuli were calibrated to meet each subject's individual pain-detection threshold. In the absence of physical alterations in stimulus input, the perception of pain can only be assessed by introspection. This means that differences in neural activity reflect different sensations of pain in response to a constant stimulation level. We found that, even when using near-threshold stimuli that make decoding maximally difficult, a ‘fingerprint’ of activity can be detected with fMRI that is sufficiently clear to enable above-chance prediction of pain perception both before and during stimulation.

### Predictive brain regions and spatial scales

In our analysis of brain regions commonly associated with the perception of pain we found that, in isolation, not all areas allowed for above-chance predictions. When decoding pain perception from pre-stimulus activity, significant accuracies were mostly afforded by the PAG and OFC (Fig. 3a). Both regions have been linked to affective processing, particularly in the context of fear and anxiety (McNally et al., 2011; Milad and Rauch, 2007). By comparison, above-chance predictions during the stimulation period were enabled by a larger set of brain regions (Fig. 3b), in particular: sensory areas (i.e., primary and secondary somatosensory cortex; Hofbauer et al., 2001; Raij et al., 2004; Singer et al., 2004); and regions involved in cognitive-affective pain processing (e.g., anterior insula; Ploner et al., 2010), including those implicated in top–down modulation of pain (i.e., DLPFC, VLPFC; Wiech et al., 2008).

An important aspect of multivariate analyses is that the significance afforded by an individual region is not equivalent to its importance in the context of a whole-brain feature space. For example, a region might provide no additional information when considered along with all other voxels in the brain, yet enable significant classification accuracies when considered in isolation. In other words, the whole-brain analysis and the region-of-interest analysis described in this paper are based on different notions of involvement; both are worthwhile studying. Thus, while one would expect a large degree of agreement between the two analyses, this need not necessarily be so. Indeed, we found that regions showing up in our whole-brain analyses (Fig. 2) typically (but not always) also afforded highly significant classification accuracies when considered in isolation in the region-of-interest analysis, and vice versa (Fig. 3). For example, while the anterior insula showed up in the map for anticipation, its accuracy was not above chance; conversely, while the left OFC did not show up in the whole-brain map for the anticipation phase, its accuracy was above chance in the region-of-interest analysis.

Our analysis of distributed activity across spatial scales showed that, both before and during stimulation, the joint activation pattern in collections of discriminative regions afforded significantly more accurate predictions than any of these regions by themselves, while being insignificantly less accurate than predictions made on the basis of whole-brain data. This result was consistent with our analysis at the scale of individual voxels. In this analysis, we found that accuracies increased quickly with the inclusion of very few initial voxels, but that this increase slowed down considerably as more and more voxels were added (Fig. 5). Taken together, these findings suggest that a multidimensional experience such as pain perception can only be understood by considering a distributed representation of activity in the brain. The highest accuracies may only be achieved when considering the entire pain matrix or indeed whole-brain activity; but most of the increase in accuracy is observed at the spatial scale of just a few regions of interest.

In summary, the multivariate analyses carried out in the present study provided two major findings that complement previous mass-univariate investigations. First, they suggest that only a subset of so-called ‘pain matrix’ regions, when considered in isolation, exhibit a distributed representation of activity across intra-areal voxels that allows for trial-wise predictions of near-threshold pain. This is in line with several recent studies in which large parts of the ‘pain matrix’ were suggested not to be pain-specific (Legrain et al., 2011; Mouraux et al., 2011). At the same time, however, our simultaneous consideration of the entire ‘pain matrix’ enabled a significantly higher prediction accuracy than the most predictive region by itself (Fig. 4b). This finding suggests that the ‘pain matrix’ as a whole carries information that might reflect the complexity of pain. As a second principal finding, our hierarchically structured analyses across multiple scales (from single regions via sets of regions to whole brain) indicate that decoding of pain perception from fMRI data is most adequately done at an intermediate spatial scale by considering the joint activity of a few core regions.

### Multivariate analyses in basic pain research and in practical applications

The results obtained in this study must be viewed in light of an important conceptual difference between cognitive neuroscience on the one hand and engineering applications, such as those found in brain–machine interfaces, on the other (cf. Friston et al., 2008). Neuroscience is typically concerned with the demonstration of structure-function relationships in the brain, which is formally carried out using model comparison (by comparing, for instance, the evidence of a model that links distributed fMRI data to pain perception to a model that does not). This means the quantity of interest is the significance with which a given prediction accuracy is above chance. Engineering applications and tools for automated diagnostics, by contrast, is typically concerned with questions of practical sufficiency (or *substantive* significance) and may therefore be more interested in, for example, the absolute classification accuracy that can be achieved in decoding a brain state from fMRI (e.g., Brodersen et al., 2011).

If one were to adopt an engineering perspective for a moment, it would be sobering to observe that even the best predictive accuracies obtained in this study were far from what could be considered satisfactory for most real-world applications. In part, this must be attributed to the fact that our near-threshold paradigm was designed to force our algorithm to rely on the subtle activity patterns encoding a purely subjective experience rather than physical differences in stimulus strength.

It is also important to keep in mind that the conclusions drawn from the application of any given statistical model (such as a general linear model or a support vector machine) are necessarily conditional on the assumptions of that model. This means that overall accuracies, the spatial deployment of informative regions, or the rank order of regions of interest are not independent of the specific characteristics of the underlying classification algorithm (such as a linear support vector machine). Thus, observing a low overall classification accuracy could in principle simply be the result of a suboptimal classification model.

The purpose of the present study, however, is not to provide a decoding algorithm that fulfills the requirements of a practically useful ‘brain-reading’ device of some sort. Instead, our ambition is to examine the distributed nature of structure-function mappings in the brain, which relies on the significance of accuracies, not on their magnitude. This is also the reason why we used the simplest and most natural classification scheme, that is, trial-by-trial classification. If one were to design an algorithm for practical prediction tasks, one might hope to achieve higher accuracies by averaging trials across several repetitions of the same trial type (Davatzikos et al., 2005).

In the general public, neuroimaging technologies are occasionally portrayed as tools that might provide an objective readout for subjective phenomena that cannot be accessed otherwise. There is indeed a pronounced need for such brain-reading tools in a legal context, where the proof of presence or absence of pain can be critical to the verdict about compensation claims (Miller, 2009). Our findings suggest that decoding of pain based on fMRI data may be possible. However, as discussed above, even the highest classification accuracies obtained in this study were a long way from perfect decoding. Furthermore, the prediction of pain for a particular individual was based on information obtained from the same individual (albeit from independent data). In a legal context, by contrast, the prediction of pain in one individual should be afforded based on information acquired in a norm collective. Although first attempts to classify across subjects have been made (e.g., Schulz et al., 2011), this approach inevitably carries the risk of neglecting individual peculiarities, especially in highly sensitive contexts such as pain perception. The potential utility of fMRI for the decoding of pain in a legal context must therefore be considered with great caution. At the same time, the underlying multivariate methods are highly useful for basic neuroscience and may eventually yield important clinical applications.

## Footnotes

2 It should be noted that the term ‘scale’ does not imply a physical scale parameter here but is used to refer to the spatial composition of the feature space, i.e., the size of the search volume used for classification.
