# Supplementary material for: Decoding the perception of pain from fMRI using multivariate pattern analysis
Source: Neuroimage. 2012 Nov 15;63(3):1162–70. doi: 10.1016/j.neuroimage.2012.08.035 (PMC3532598; doi:10.1016/j.neuroimage.2012.08.035)
Supplement: Supplementary file 1 — Supplementary material. [file mmc1.pdf]

SUPPLEMENTAL MATERIAL

Decoding the perception of pain from fMRI using multivariate pattern analysis

Brodersen*, Wiech*, Lomakina, Lin, Buhmann, Bingel, Ploner, Stephan, Tracey

A Supplemental figures

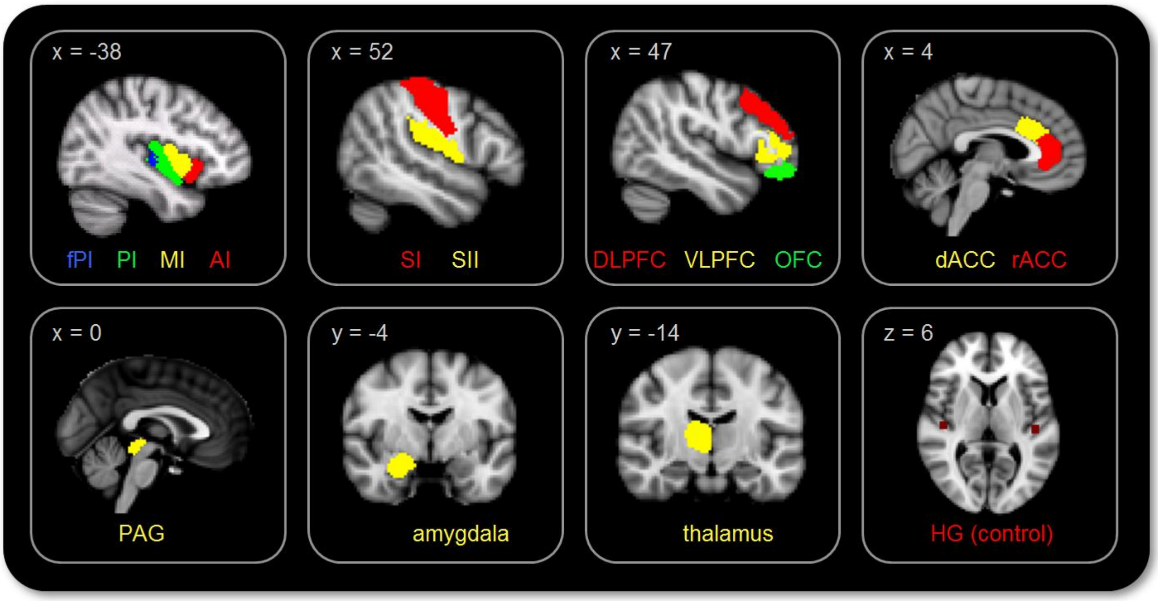

Fig. S1 | Regions of interest. For a detailed definition of the ROIs see *Information on anatomical regions of interest* (Section B).

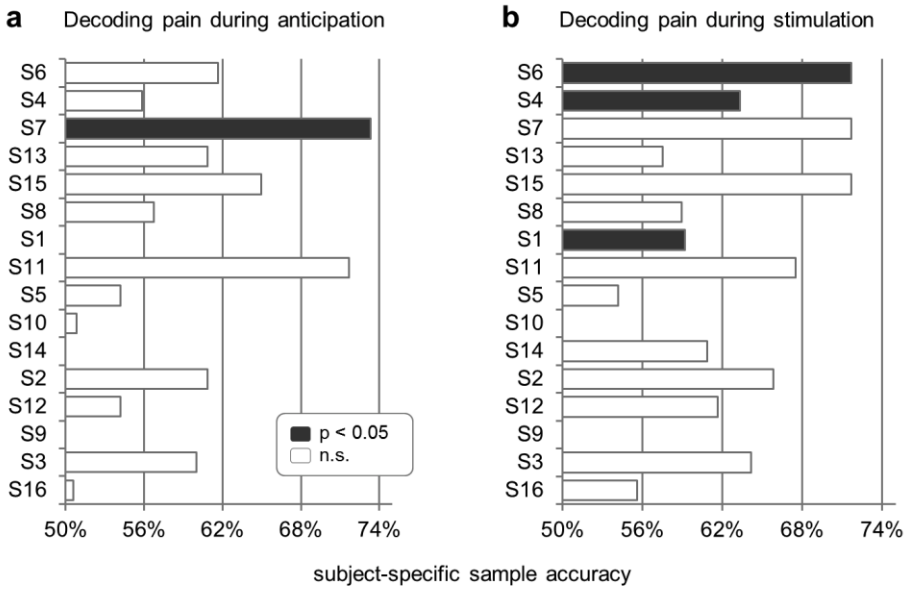

Fig. S2 | Subject-specific classification results. This figure shows subject-specific classification accuracies in predicting the subjective perception of pain (a) *before* and (b) *during* stimulation (120 trials per subject). Results are given in terms of sample accuracies (number of correctly classified trials divided by number of trials). Statistical significance was evaluated using a nonparametric permutation test (N = 1000) with Bonferroni correction for multiple-testing (16 subjects). Subjects are ordered in descending order by the product of the p-values in (a) and (b).

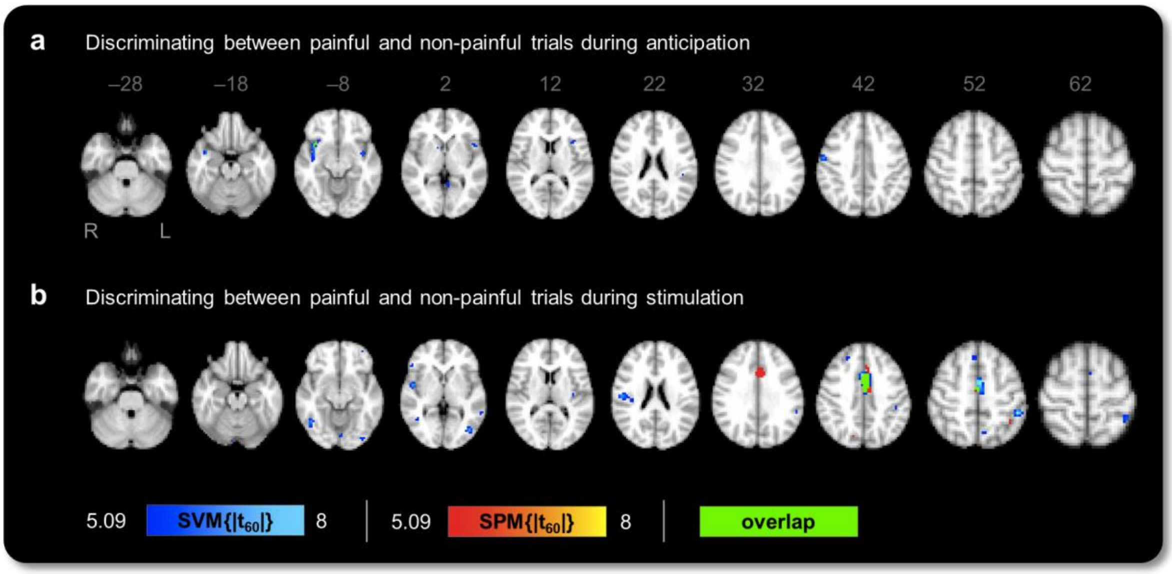

Fig. S3 | Comparison of SVM and SPM results. Axial slices show the results of a (multivariate) support vector machine (SVM) discriminative map (blue) and the corresponding (mass-univariate) whole-brain statistical parametric map (SPM, red). Both maps are based on a two-tailed contrast between subjectively painful and non-painful trials (16 subjects). Separate analyses were conducted based on brain activity (a) *before* and (b) *during* stimulus application. Voxels identified by both methods are highlighted (green). Significant voxels are overlaid onto a standard structural scan in MNI152 space. Results from both methods are given in terms of absolute *t*-scores on 60 degrees of freedom ($p < 0.05$, FWE-corrected).

B Information on anatomical regions of interest

Dorsal and rostral anterior cingulate cortex (dACC and rACC). The mask for the dorsal ACC corresponds to the anterior midcingulate cortex (Brodman area 24a’, b’, c’, and 32’), while the rACC was defined as the perigenual ACC (area 24a, b, c, and 32), following the division proposed in the literature (Vogt, 2005).

Primary and secondary somatosensory cortex (SI and SII). The mask for SI occupies the posterior bank of the central sulcus and the postcentral gyrus, and extends from the dorsal surface of the brain to the lateral fissure corresponding to Brodmann areas 1, 2, 3a, and 3b. The mask for SII was drawn over the superior bank of the posterior insula, where it can be viewed in the sagittal section, and continued in the coronal section over the superior bank of the lateral fissure starting from the rostralmost slice corresponding to the plane of the line passing through the anterior commissure (Eickhoff, Schleicher, Zilles, & Amunts, 2006).

Dorsolateral and ventrolateral prefrontal cortex (DLPFC and VLPFC). The mask for the DLPFC comprises BA8, 9, 46, and 9/46 located in the superior and middle frontal gyri (Petrides, 2005). The VLPFC was defined

as Brodman areas 44 (pars opercularis), 45 (pars triangularis), and the lateral part of area 47/12 of the inferior frontal gyrus (Petrides & Pandya, 2002; Petrides, 2005).

Amygdala. The amygdala lies in the superomedial aspect of the mesial temporal lobe (Amunts et al., 2005). It gradually enlarges posteriorly and decreases in size when in contact with the hippocampus. The transition slice where the hippocampus first appears on the more posterior slices and borders the amygdala inferomedially is identified by white matter tracts on the medial border of the hippocampus, which appears as a line of gray matter curving superomedially.

Orbitofrontal cortex (OFC). The OFC mask occupies Brodmann areas 10, 11, 12, 13, 14, and the orbital part of area 47/12 (Kringelbach, 2005; Petrides & Pandya, 2002). It extends from the horizontal ramus of the lateral fissure on the lateral surface to the orbital surface and onto the medial surface to include the gyrus ventral to cingulate sulcus and the subcallosal cingulate areas. The boundary on the medial surface is from the rostral sulcus to the horizontal ramus of the lateral fissure (Croxson et al., 2005).

Anterior, mid, and posterior insula. The insula was divided into an anterior, mid, and posterior division (Jonathan C W Brooks, Nurmikko, Bimson, Singh, & Roberts, 2002). The mask for the anterior insula includes tissue from the anterior gyrus brevis, the mid insula comprises the posterior gyrus brevis, and the posterior insula was defined as the anterior gyrus longus. For the purposes of an additional *post-hoc* analysis (see Results), an independent dataset was used (Brooks, Zambreanu, Godinez, Craig, & Tracey, 2005) to define a somatotopically informed functional mask of the posterior insula.

Thalamus. The thalamus lies between the foramen of Monro and the posterior commissure and extends from the third ventricle medially to the medial border of the posterior limb of the internal capsule laterally (Ooteman and Cretsinger, Thalamus tracing guidelines: www.psychiatry.uiowa.edu/mhcrc/pdf/papers/thalamus.pdf). Throughout its course it maintains a close relationship with the internal capsule on the superolateral aspect. Its visualization ends just beyond the level of the corpora quadrigemina. The mask for the thalamus was drawn in the coronal view starting from the rostral aspect, at the plane where the anterior commissure can be visualized maximally, and moving caudally.

PAG. The periaqueductal grey surrounds, as its name implies, the aqueduct of sylvius in the midbrain (Naidich, 2009). The outline of PAG was first delineated in the sagittal view, and the mask drawn in the axial view according to the Duvernoy's atlas of the human brainstem (Naidich, 2009).

Grey-matter control masks of regions not involved in pain processing. The grey-matter regions were defined as a cubic area of 4x4x4 voxels within primary auditory cortex (Heschl's gyrus, HG).

C Synopsis of classification analyses

Whole-brain map discriminative map

- Preprocessing (smoothing with conventional FWHM 8 mm)
- Repeat for all subjects
    - Train a linear SVM on all voxels in the brain
    - Reconstruct voxel-specific weights
- Compute average voxel weights (across subjects)
- Loop over *N* = 2000 permutations
    - Repeat for all subjects
        - Randomly permute class labels
        - Train a linear SVM on all voxels in the brain
    - Compute average voxel weights (across subjects)
- For each voxel, estimate mean and variance of null distribution
- Compute map of *t*-scores

Whole-brain / ROI-based classification analyses

- Preprocessing (smoothing with smaller FWHM 5 mm)
- Repeat for all subjects
    - Leave-one-session-out cross-validation
        - Using the current training data (i.e., all trials from 3 out of 4 sessions)
            - Linear search over hyperparameter *C*
            - Nested leave-one-trial-out cross-validation to assess the performance afforded by each hyperparameter setting
        - Train a linear SVM on all voxels in the given mask, using the optimal hyperparameter found above
        - Test the SVM on the trials from the left-out session
    - Compute sample classification accuracy (number of correctly classified trials divided by number of trials in total)
- Compute mean classification accuracy across all subjects
- Loop over *N* = 1000 (whole-brain) or *N* = 2600 (ROI-based) permutations
    - Repeat for all subjects
        - Permute class labels within sessions
        - Leave-one-session-out cross-validation

            - Using the current training data (i.e., all trials from 3 out of 4 sessions)
                - Linear search over hyperparameter *C*
                - Nested leave-one-trial-out cross-validation to assess the performance afforded by each hyperparameter setting
            - Train a linear SVM on all voxels in the given mask, using the optimal hyperparameter found above
            - Test the SVM on the trials from the left-out session
        - Compute sample classification accuracy (number of correctly classified trials divided by number of trials in total)
    - Compute mean classification accuracy across all subjects
- Obtain *p*-value from a nonparametric permutation test on classification accuracies

Classification analyses based on most discriminative voxels

- Preprocessing (smoothing with smaller FWHM 5 mm)
- Repeat for all subjects
    - Leave-one-session-out cross-validation
        - Compute voxel-specific discriminative score using a two-tailed *t*-test
        - Select top *k* voxels (in terms of largest absolute *t*-scores)
        - Using the current training data and selected *k* voxels
            - Linear search over hyperparameter *C*
            - Nested leave-one-trial-out cross-validation to assess the performance afforded by each hyperparameter setting
        - Train a linear SVM on selected *k* voxels, using the optimal hyperparameter found above
        - Test the SVM on the trials from the left-out session
    - Compute sample classification accuracy (number of correctly classified trials divided by number of trials in total)
- Compute mean classification accuracy across all subjects
- Loop over *N* = 1000 permutations
    - Repeat for all subjects
        - Permute class labels within sessions
        - Leave-one-session-out cross-validation
            - Compute voxel-specific discriminative scores using a two-tailed *t*-test
            - Select top *k* voxels (in terms of largest absolute *t*-scores)

            - Using the current training data and selected *k* voxels
                - Linear search over hyperparameter *C*
                - Nested leave-one-trial-out cross-validation to assess the performance afforded by each hyperparameter setting
            - Train a linear SVM on selected *k* voxels, using the optimal hyperparameter found above
            - Test the SVM on the trials from the left-out session
        - Compute sample classification accuracy (number of correctly classified trials divided by number of trials in total)
    - Compute mean classification accuracy across all subjects
- Obtain *p*-value from a nonparametric permutation test on classification accuracies

Supplemental references

Amunts, K., Kedo, O., Kindler, M., Pieperhoff, P., Mohlberg, H., Shah, N. J., Habel, U., et al. (2005). Cytoarchitectonic mapping of the human amygdala, hippocampal region and entorhinal cortex: intersubject variability and probability maps. *Anatomy and Embryology*, *210*(5-6), 343-352. doi:10.1007/s00429-005-0025-5

Brooks, J C W, Zambreanu, L., Godinez, A., Craig, A. D. B., & Tracey, I. (2005). Somatotopic organisation of the human insula to painful heat studied with high resolution functional imaging. *NeuroImage*, *27*(1), 201-209. doi:10.1016/j.neuroimage.2005.03.041

Brooks, Jonathan C W, Nurmikko, T. J., Bimson, W. E., Singh, K. D., & Roberts, N. (2002). fMRI of thermal pain: effects of stimulus laterality and attention. *NeuroImage*, *15*(2), 293-301. doi:10.1006/nimg.2001.0974

Croxson, P. L., Johansen-Berg, H., Behrens, T. E. J., Robson, M. D., Pinsk, M. A., Gross, C. G., Richter, W., et al. (2005). Quantitative investigation of connections of the prefrontal cortex in the human and macaque using probabilistic diffusion tractography. *The Journal of Neuroscience: The Official Journal of the Society for Neuroscience*, *25*(39), 8854-8866. doi:10.1523/JNEUROSCI.1311-05.2005

Eickhoff, S. B., Schleicher, A., Zilles, K., & Amunts, K. (2006). The human parietal operculum. I. Cytoarchitectonic mapping of subdivisions. *Cerebral Cortex (New York, N.Y.: 1991)*, *16*(2), 254-267. doi:10.1093/cercor/bhi105

Kringelbach, M. L. (2005). The human orbitofrontal cortex: linking reward to hedonic experience. *Nature Reviews. Neuroscience*, *6*(9), 691-702. doi:10.1038/nrn1747

Naidich, T. P. (2009). *Duvernoy's atlas of the human brain stem and cerebellum*. New York: Springer-Verlag.

Petrides, M, & Pandya, D. N. (2002). Comparative cytoarchitectonic analysis of the human and the macaque ventrolateral prefrontal cortex and corticocortical connection patterns in the monkey. *The European Journal of Neuroscience*, *16*(2), 291-310.

Petrides, Michael. (2005). Lateral prefrontal cortex: architectonic and functional organization. *Philosophical Transactions of the Royal Society of London. Series B, Biological Sciences*, *360*(1456), 781-795. doi:10.1098/rstb.2005.1631

Vogt, B. A. (2005). Pain and emotion interactions in subregions of the cingulate gyrus. *Nature Reviews. Neuroscience*, *6*(7), 533-544. doi:10.1038/nrn1704
